# Use of Mogamulizumab for Cutaneous Adult T-cell Leukemia in a Patient Living With HIV

**DOI:** 10.7759/cureus.31701

**Published:** 2022-11-20

**Authors:** Stephanie Boisclair, Amanda Brahim, Jose Sandoval-Sus

**Affiliations:** 1 Internal Medicine, Memorial Healthcare System, Pembroke Pines, USA; 2 Hematology/Oncology Pharmacy, Memorial Cancer Institute, Pembroke Pines, USA; 3 Oncology and Hematology, Moffitt Malignant Hematology & Cellular Therapy at Memorial Healthcare System, Pembroke Pines, USA

**Keywords:** hiv, human immunodeficiency virus, human immunodeficiency virus infection, adult t cell lymphoma, mogamulizumab, clinical trial disparities, htlv-1/2

## Abstract

Human T-lymphotropic virus type 1 (HTLV-1) is known to cause a rare form of leukemia/lymphoma called adult T-cell leukemia/lymphoma (ATLL). Although ATLL is known to have a high co-infection rate with human immunodeficiency virus (HIV) in areas where both viruses are endemic, clinical trials, such as the phase three trial for mogamulizumab, continue to exclude patients living with HIV. We here describe the utilization and therapeutic course of mogamulizumab for ATLL in a patient living with HIV. Unfortunately, due to exclusion of patients with co-viral infections in trials, decisions regarding clinical care in these patients remain challenging with the need to rely on retrospective publications for safety and efficacy.

## Introduction

Human T-lymphotropic virus type 1 (HTLV-1) is known to cause a rare form of non-Hodgkin's lymphoma called adult T-cell leukemia/lymphoma (ATLL) - a neoplasm of CD4+ T-cells. HTLV-1 is predominantly known in the Caribbean, Asian, African, and Hispanic populations with a known HIV co-infection rate as high as 28% in areas where both viruses are endemic [1,2]. Although there is no standard of care, treatment options include interferon-γ with zidovudine, chemotherapy (i.e. cyclophosphamide, hydroxydaunorubicin, oncovin, and prednisone (CHOP)), and histone deacetylase (HDAC) inhibitors. Recently, mogamulizumab (mog) was approved for relapsed cutaneous T-cell lymphoma (CTCL) after the phase 3 MAVORIC trial showed improved progression-free survival and quality of life compared to vorinostat [3]. Mog, a monoclonal antibody against C-C chemokine receptor-4 (CCR4), inhibits the migration and proliferation of T-cells while also initiating the antibody-dependent cellular cytotoxicity (ADCC) pathway [3]. CCR4 expression is universally present in ATLL with a gain of function mutation identified in 26-33% of cases [4]. Patients living with HIV (PLWH) were excluded from the MAVORIC trial and all mog trials thereafter. Hence, its safety and effectiveness remain unknown in this population.

## Case presentation

A 60-year-old black man from Haiti presented to the clinic with a pruritic, papular rash on the lower abdomen and back. He had a complex medical history including systolic heart failure (American Heart Association (AHA) stage B), chronic kidney disease, and HIV on antiretroviral therapy (emtricitabine-tenofovir alafenamide and raltegravir). He was diagnosed with smoldering ATLL in 2013 with previous treatment of CHOP, interferon-γ, and romidepsin. At first encounter, he had an undetectable HIV viral load and a CD4+ count of 1609 cells/uL. A cutaneous punch biopsy of a papule confirmed cutaneous ATLL, and further diagnostic workup, including imaging, bone marrow biopsy, and peripheral flow cytometry, was negative for systemic involvement.

**Figure 1:**
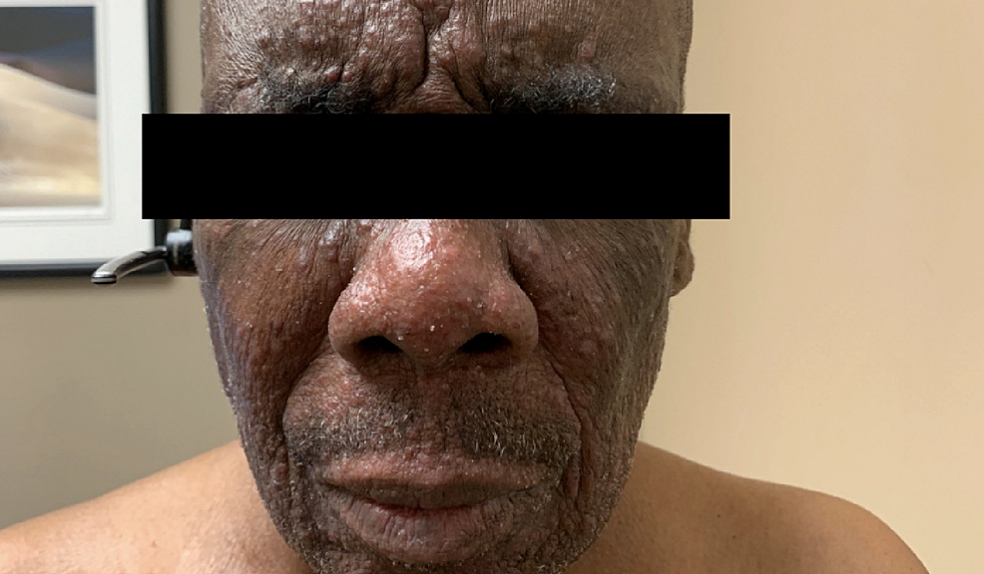
Cutaneous lesions on the face

**Figure 2:**
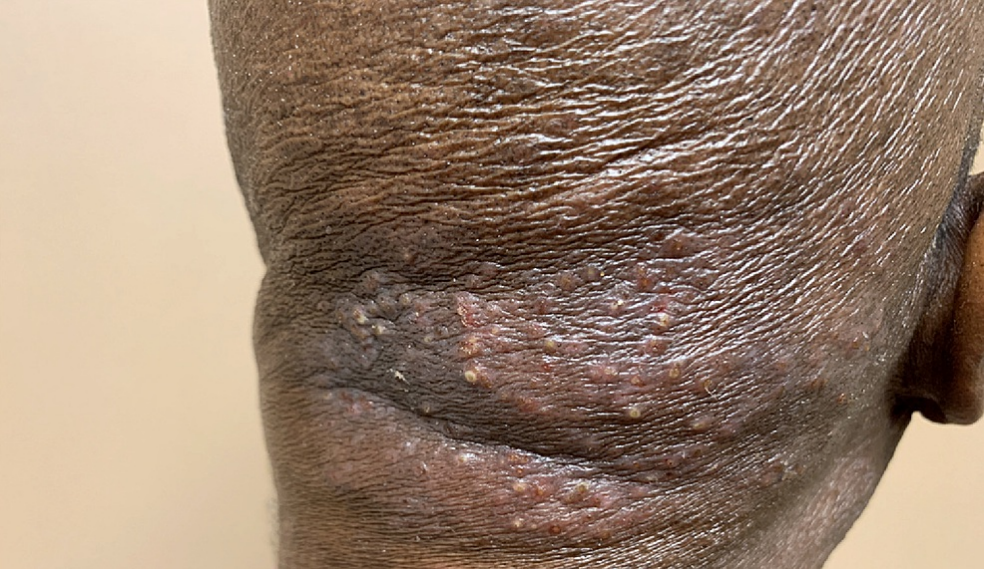
Cutaneous lesions on the back of the neck

After six months of treatment with vorinostat and topical triamcinolone, his disease progressed with facial and cervical lesions, associated with severe pruritus (Figures 1, 2).

An increasing number of polylobated lymphocytes with neoplastic appearance, also known as “flower” cells, were found in peripheral blood. At progression, the patient had an undetectable HIV viral load with a CD4+ count of 5293 cells/uL. Taking into consideration the patient’s multiple comorbidities, mog was initiated at the standard FDA-approved dose for CTCL [3].

**Figure 3:**
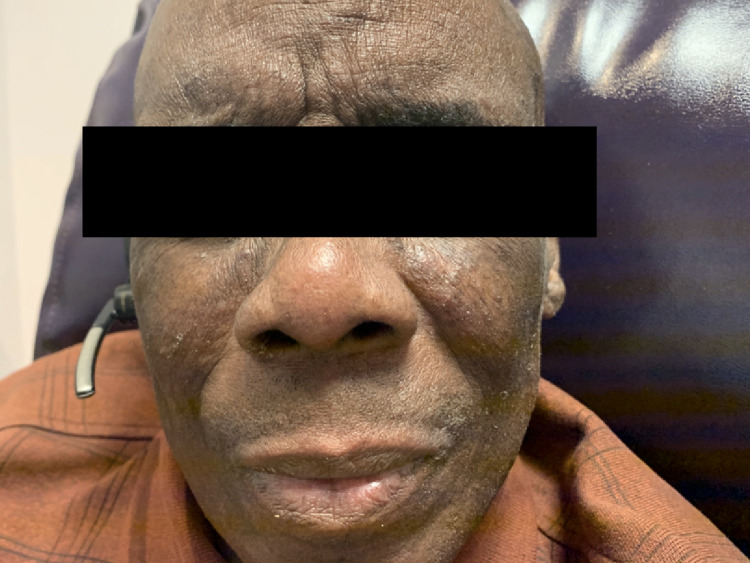
Resolution of cutaneous lesions on the face after one month of treatment

**Figure 4:**
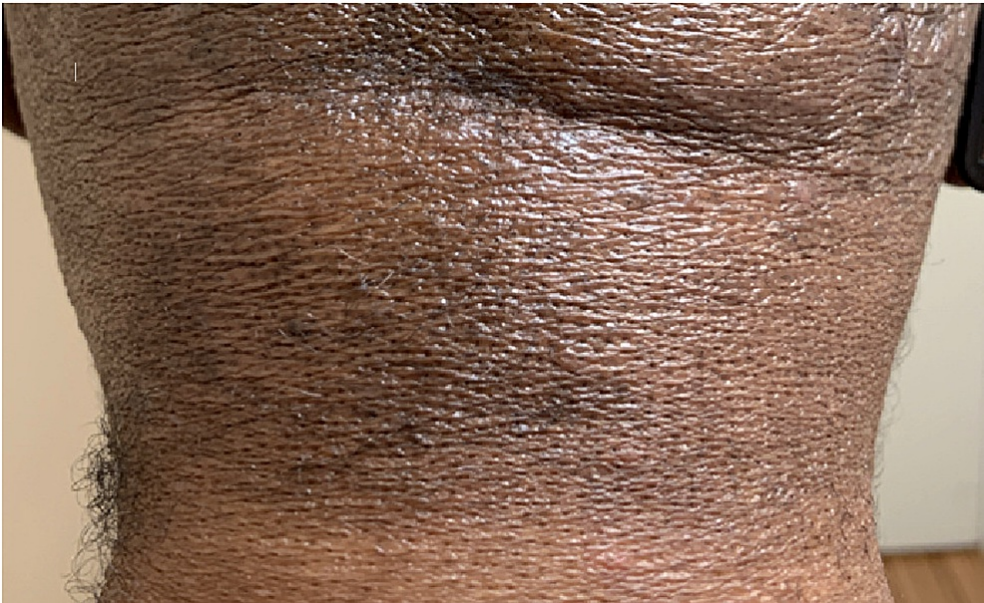
Resolution of the back of neck lesions after one month of treatment

Rapid, near-to-complete resolution of skin lesions occurred within the first cycle (Figures 3, 4).

He tolerated 11 cycles of mog with only grade 1 anemia. HIV viral load remained undetectable with CD4+ counts decreasing to a plateau of 510 cells/uL (coinciding with ATLL remission). The patient remains alive and is still receiving mog therapy, with continued disease control.

## Discussion

Mog, a humanized IgG1 kappa monoclonal antibody, selectively binds to CCR4 and initiates antibody-dependent cellular cytotoxicity resulting in target cell depletion [3]. CCR4 is expressed in a variety of cells including T-helper cells and T-regulatory cells. It is also consistently expressed in T-cell malignancies, making it a potential therapeutic target for such diseases. However, significant comorbidities where lymphocytes are already at risk of depletion, such as HIV co-infection, create a clinical dilemma for its use.

Although first approved in Japan for ATLL in 2012, mog was approved by the FDA in 2018 for CTCLs based on the MAVORIC phase 3 trial, which showed a doubling of progression-free survival vs. vorinostat (7.7 months vs. 3.1 months) as well as improved overall response rate [3]. The patient population included in this study was heavily pretreated and received a median of three prior treatments, similar to our patient. However, this trial specifically excluded PLWH although the co-infection of these viruses is well known. There are no known pharmacokinetic interactions between mog and antiretrovirals, mainly secondary to the lack of reports analyzing this relationship. Furthermore, there are no available studies discussing the use of mog in PLWH. We hypothesized mog would be effective and safe because the ADCC pathway and the CCR4 receptor have been shown to remain active in HIV-infected cells. However, with known consequences of severe infections and immune-mediated complications associated with mog [3], we emphasized judicious HIV control throughout treatment. To our knowledge, this is the first report demonstrating the safety and efficacy of mog in a cutaneous ATLL PLWH.

Several prior studies suggested the unreliability of CD4+ counts in monitoring immune reconstitution for PLWH co-infected with HTLV-1, as it induces clonal proliferation of ineffective CD4+ lymphocytes [2]. Our patient reproduced this trend with an abnormally elevated CD4+ cell count at progression followed by a CD4+ count downtrend and normalization with ATLL response. Prior case reports have discussed the implication of HIV’s lymphocyte-depleting pathophysiology correlating with an indolent ATLL presentation in co-infected patients [5]. HIV infection may play a role in the prolonged ATLL disease control in our patient who continues to have an unexpectedly prolonged overall survival. This is especially important since patients with relapsed/refractory ATLL typically have poor response rates to single-agent mog [6]. This case presentation demonstrates the importance of considering co-morbidities, such as HIV, and their relation to overall survival in cancer. Further prospective studies of mog in ATLL PLWH should be considered to establish safety and improved overall survival in this minority subgroup.

## Conclusions

This case describes the safety profile and treatment response of mog in a patient living with HIV and cutaneous smoldering ATLL. It is imperative that clinical trials include patients with significant co-morbidities seen in the real-world population. Not only would this prevent ascertainment bias, but it would also enhance the external validity of approved treatments and reduce treatment dilemmas in PLWH. Publications discussing outcomes of PLWH treated with immunotherapy outside clinical trials remain important due to the unmet needs of this population.
